# Calcific tendinosis of flexor carpi radialis as a cause of wrist pain

**DOI:** 10.1093/jscr/rjae751

**Published:** 2024-12-01

**Authors:** Kim Calleja Urry, Kurstein Nicholas Sant, John Anthony Casaletto

**Affiliations:** Department of Orthopaedics, Trauma and Sports Medicine, Mater Dei Hospital, Dun Karm Street, Msida, MSD 2090, Malta; Department of Orthopaedics, Trauma and Sports Medicine, Mater Dei Hospital, Dun Karm Street, Msida, MSD 2090, Malta; Department of Orthopaedics, Trauma and Sports Medicine, Mater Dei Hospital, Dun Karm Street, Msida, MSD 2090, Malta

**Keywords:** case report, calcific tendinosis, wrist pain, flexor carpi radialis

## Abstract

Calcific tendinosis is characterized by the deposition of calcium hydroxyapatite crystals within the substance of the tendon. We present a case of a 52-year-old female who presented with a 6-month history of right wrist pain, along with a palpable and tender lump in the region of the flexor carpi radialis tendon at the level of the distal radius. Radiographs confirmed the presence of calcific tendinosis of the flexor carpi radialis tendon. The patient was referred for hand therapy sessions and was prescribed nonsteroidal anti-inflammatory drugs, which led to a gradual improvement after 4 months. A repeat radiograph confirmed the resolution of the previous calcific focus at the flexor carpi radialis tendon region. This case highlights the importance of considering rare causes of wrist pain such as this case. Various treatment modalities are available, including surgical management, but ultimately, treatment should be within a multidisciplinary team environment.

## Introduction

Wrist pain is one of the most common complaints encountered in Orthopedics clinical practice. Calcific tendinosis of flexor carpi radialis represents one of the rarer etiologies of wrist pain [1]. This condition is characterized by the deposition of calcium hydroxyapatite crystals within the tendon, most commonly affecting adults between 30 and 60 years of age [2]. Calcific tendinosis commonly affects joints such as the shoulders, and its occurrence in the wrist is not frequently reported in the literature [1]. This case emphasizes the importance of considering less common pathologies as part of the differential diagnosis for wrist pain and aims to aid the clinician in early recognition and management of this condition.

## Case report

The patient has provided informed consent to participate in this case report. This case concerns a 52-year-old female who presented because of a 6-month history of right wrist pain. The patient denied a history of trauma and noted that pain was worse with activities and was also present at night.

The patient had a medical history relevant to hypertension and hyperlipidemia and was allergic to penicillin. Her surgical history was relevant for a left shoulder arthroscopy done in 2018 to remove calcification in the left supraspinatus tendon, causing persistent left shoulder pain. She also had similar symptoms in the right shoulder, which developed in 2021. An X-ray of the right shoulder also confirmed the presence of calcification at the region of the right supraspinatus tendon, but this was not managed surgically.

**Figure 1:**
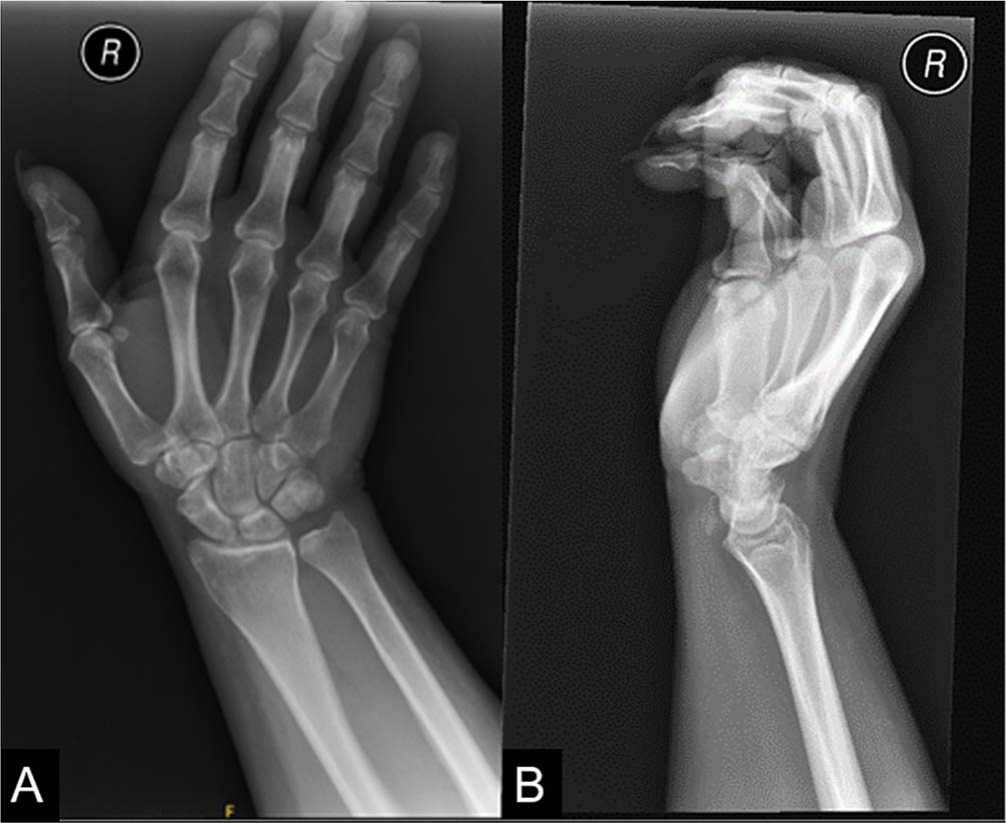
An X-ray of the right wrist which shows calcification at the level of flexor carpi radialis at the wrist.

On examination of the right wrist, the patient had a palpable, tender lump in the region of the flexor carpi radialis tendon at the level of the distal radius. There was no associated erythema. Given the pain, the patient had a reduced range of movement during volar flexion of the wrist. There were no abnormalities during a neurovascular examination. Blood investigations were all unremarkable. An X-ray of the right wrist confirmed calcification at the flexor carpi radialis level at the wrist (Fig. 1).

**Figure 2:**
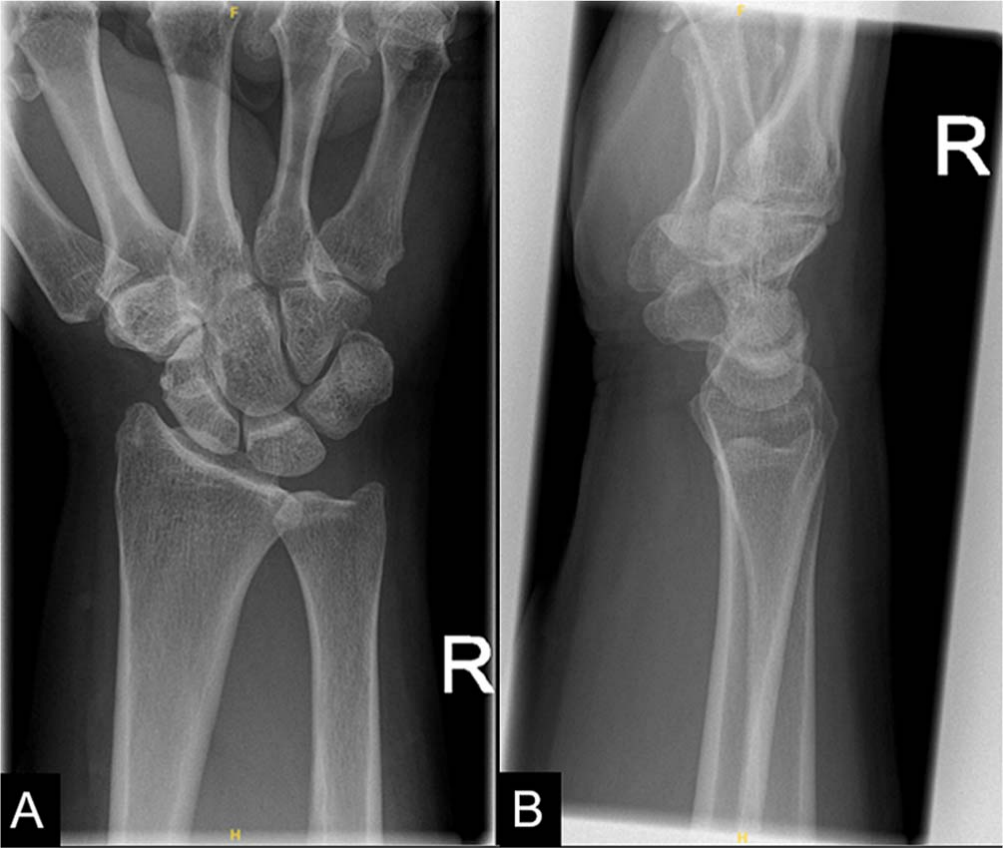
A repeat X-ray of the right wrist indicating that the previous calcific focus at the flexor carpi radialis tendon region had resolved.

The patient was referred for hand therapy sessions and was prescribed non-steroidal anti-inflammatories to alleviate the pain. She was regularly being followed up in the clinic, where her symptoms had drastically improved after 4 months. A repeat X-ray of her right wrist showed that the previous calcific focus at the flexor carpi radialis tendon region had resolved following conservative management (Fig. 2).

## Discussion

Calcific tendinosis of the hand is an uncommon condition, first described by Cohen in 1924 with deposition of calcium at flexor carpi ulnaris insertion following trauma [3]. Thus, the unusual nature of this condition may still lead to underrecognition and misdiagnosis [1].

Uhthoff and Loehr [4] have proposed that the progression of calcific tendinopathy consists of three phases: precalcific, calcific, and postcalcific. In the precalcific phase, collagen fibers undergo metaplastic change into fibrocartilage tissue in response to the changes in the metabolic and mechanical conditions of the tendon. The formative, resting, and resorptive stages are different components of the calcific phase. During the formative stage, chondrocytes develop within areas of fibrocartilage formation, resulting in calcified apatite crystals. The calcified area then enters a resting state. However, it commonly progresses to the resorptive stage, characterized by an inflammatory process with the appearance of leukocytes, lymphocytes, and giant cells, which form a “calcium granuloma” [4]. The resorptive stage is the phase that causes most debilitation in patients, as seen typically in the shoulder, where calcium crystals extravasate in the subacromial bursa may cause severe pain and restriction of movement [5]. The calcification then enters the postcalcific phase, where fibroblasts remodel the tendon tissue, and granulation tissue replaces calcium deposits [4, 5]. This cycle is only sometimes followed and may be blocked at any point by different causes. In addition, the relatively poor tendon vascularity may also contribute to the failure of the structure’s self-healing capacity [6].

Presentation is typically with chronic pain in the affected joint, but findings may also be incidental. Calcific tendinosis most commonly presents in the shoulder but can also present in areas such as the wrists [1, 7]. Kwiecien *et al*. [7] reported a case of calcific tendonitis at the proximal myotendinous junction of flexor carpi radialis, diagnosed in a throwing athlete with chronic pain in the forearm. The patient had failed conservative management and underwent surgical excision of the calcified tendon [7]. Kim and Park [8] also reported thirty cases of acute calcium deposition in the hands and wrists, which were all managed with nonsteroidal anti-inflammatories or steroid infiltrations. Thirteen of these patients presented with acute calcific peritendinitis, where flexor carpi ulnaris was affected in 12 cases, and extensor carpi ulnaris was affected in 1 case. The other 17 cases of acute calcific periarthritis mainly affected the metacarpophalangeal and interphalangeal joints. Interestingly, the authors noted that four patients with acute calcific peritendinitis experienced a recurrence of symptoms with the persistence of calcifications on follow-up imaging. In contrast, no patients from the other group experienced further pain [8].

Although many cases of calcific tendinosis resolve spontaneously, several remain symptomatic, leading to the theory of halting the natural cycle of resorption of the calcium deposition, as indicated by follow-up imaging showing the persistence of calcification [6]. Several treatment methodologies have been proposed, including conservative management with rest, use of nonsteroidal anti-inflammatory medications, hand therapy, and corticosteroid infiltrations. More invasive options should be considered when conservative management fails to alleviate symptoms. Extracorporeal shock wave therapy has also shown benefits in cases of calcific tendinosis [9]. The proposed mechanism of action is the induction of the early release of angiogenic and proliferating growth factors, which positively influence the neovascularization of the tendon and may aid in reactivating the regeneration potential [10]. Alternatively, surgical excision of the tendon’s calcified section is also proposed [7].

In conclusion, this case report sheds light on the rare diagnosis of calcific tendinosis of flexor carpi radialis as a potential cause of wrist pain. Various treatment modalities can be considered, but management should be tailored individually and within a multidisciplinary team comprising the orthopedic surgeon, radiologists, and physical therapist to achieve the best outcome for the patient.

## Acknowledgements

We would like to extend our gratitude to Mater Dei Hospital, Malta, for granting us the opportunity to carry out this case report, enabling us to contribute to medical knowledge while benefiting from the hospital’s facilities, support, and expertise. We have no other disclaimers to declare regarding our article.

## Conflict of interest statement

None declared.

## Funding

None declared.
